# Letting happiness happen

**DOI:** 10.1016/j.eclinm.2025.103467

**Published:** 2025-08-22

**Authors:** eClinicalMedicine

Often overlooked amid seasonal shifts and holiday planning, August is quietly dedicated to one of the most powerful human emotions—happiness—and is recognised as Happiness Happens Month. The core of this event is a simple yet profound idea—happiness is contagious, it matters, and is worth celebrating. Happiness Happens Month was started by the Society of Happy People in 1999, when the society declared August as the month to recognise and express happiness. The organisation was founded by Pamela Gail Johnson in 1998 with a mission to encourage people to share their happiness without guilt or fear of judgment. Initially, Aug 8 was designated as “Admit You're Happy Day”, but the celebration soon expanded to encompass the entire month due to the overwhelmingly positive response, and 25 years later, the message is just as strong and important.

The world today is not an easy place to thrive in. People can be directly or indirectly affected by stress, deadlines, conflict, turmoil, and mental and physical exhaustion, and intentionally pausing to recognise happiness might seem trivial or even indulgent. Scientifically, we have yet to come to a consensus of what happiness is, an interplay of neurotransmitters, genetics, environment, and personal circumstances. However, research shows us that happiness is more than just a nice feeling; it is a key indicator of wellbeing, resilience, and social connection. Aristotle once said ‘‘Happiness is the meaning and purpose of life, the whole aim and the end of human existence”, and today we have the science to support the claim.

How does happiness affect our health? To begin with, happiness promotes a healthy lifestyle. Studies have shown that those with positive thoughts and wellbeing are more likely to consume healthier foods, such as fresh fruits and vegetables, than are those with a lower psychological wellbeing. They are also likely to be more physically active. Happiness boosts our immune system and reduces stress levels. It also protects the heart. A 10-year longitudinal study of 1500 adults showed that even in the presence of risk factors, such as older age, high cholesterol levels, and hypertension, those with more positive emotions and happiness were associated with a 22% lower risk of developing heart disease than those who did not report being happy. Another study has shown that happiness can reduce the perception and intensity of pain, and increase our tolerance to it.

The phrasing Happiness Happens is intentional. It suggests the notion that happiness is not always something we can or should chase—it is something we notice, create, or allow. It happens every day and all around us. Happiness, like any aspect of a healthy life, is something we need to keep working on, and there are ways we can give ourselves the positive boost we need. The UK National Health Service offers tips on how to be happier, more in control of our emotions, and cope better with life's challenges.

One way to improve happiness is by adopting a healthier lifestyle, which could include increasing physical activity, eating a healthier diet, focusing on getting a good night's sleep, and spending more time in nature. A study conducted in Finland, reported to be “the happiest country in the world”, showed that participants agreed that nature makes them happy or associated only positive feelings with nature. Additionally, nature experiences have been shown to reduce stress and, in an urban environment, access to green spaces can benefit children's cognitive and motor development. Long-term exposure to residential greenness in the UK has been linked to a decreased risk of incident depression and anxiety. The concept of ‘Happy Urban public spaces’ shows how nature has a profound positive effect on resident wellbeing. The type of physical activity also matters. A study of adults in Lithuania showed that physical activity associated with leisure had more health benefits than physical activity associated with work. So, go for that stroll in the park. As a matter of fact, many of London's parks were established during the Victorian era as a public health measure, aiming to improve the physical and mental wellbeing of the city's residents.

Another factor to happy and healthy living, which is often overlooked, is human connection. The Harvard Study of Adult Development, one of the longest running studies on happiness (85 years) shows us that positive relationships keep us happier and healthier than do career achievements, money, fame, and success. Social fitness is just as important as physical or mental fitness. In our ever-busy lives, we tend to ignore established relationships, forgetting that they too need our attention and social fitness needs its own exercise. Take the time to call your old friends, have a heart-to-heart with your partner away from the shadow of your digital devices, perform random acts of kindness, or volunteer for a personal cause.

Giving yourself the time to relax and focus on self-care is important. Happiness is not perfection, it is normal to have bad days, and there is no shame in asking for help. Letting happiness in where we can, even on tough days, is what matters. Small actions can help us to incorporate positive emotions and happiness in our lives: starting a gratitude journal, swapping screen time for time outdoors, listening to music, or picking up a new hobby.

Acknowledging happiness in a structured way, such as during Happiness Happens Month, is not just symbolic—it can be transformative. When individuals focus on happiness, they become healthier, communities become stronger, workplaces become more collaborative, and families become more supportive. Let us collectively think of August as a breather—a space to laugh more freely, love more deeply, and live more intentionally. This August, let happiness happen. Notice it. Celebrate it. Share it.

